# LMTK3 confers chemo-resistance in breast cancer

**DOI:** 10.1038/s41388-018-0197-0

**Published:** 2018-03-15

**Authors:** Justin Stebbing, Kalpit Shah, Lei Cheng Lit, Teresa Gagliano, Angeliki Ditsiou, Tingting Wang, Franz Wendler, Thomas Simon, Krisztina Sára Szabó, Timothy O’Hanlon, Michael Dean, April Camilla Roslani, Swee Hung Cheah, Soo-Chin Lee, Georgios Giamas

**Affiliations:** 1 Department of Surgery and Cancer, Division of Cancer, Imperial College London, Hammersmith Hospital Campus, Du Cane Road, London, W12 ONN UK; 2 Division of Cancer Epidemiology and Genetics, National Cancer Institute (NCI), National Institutes of Health (NIH), Bethesda, MD 20892 USA; 3 Department of Physiology, Faculty of Medicine, University of Malaya, 50603 Kuala Lumpur, Malaysia; 4 School of Life Sciences, Department of Biochemistry and Biomedicine, University of Sussex, Brighton, BN1 9QG UK; 5 Cancer Science Institute of Singapore, Centre for Life Sciences, 28 Medical Drive, #02-15, Singapore, Singapore; 6 Cancer Genomics Research Laboratory, National Cancer Institute, Division of Cancer Epidemiology and Genetics, Leidos Biomedical Research Inc., Bethesda, MD 20892 USA; 7 Department of Surgery, Faculty of Medicine, University of Malaya, 50603 Kuala Lumpur, Malaysia

## Abstract

Lemur tyrosine kinase 3 (LMTK3) is an oncogenic kinase that is involved in different types of cancer (breast, lung, gastric, colorectal) and biological processes including proliferation, invasion, migration, chromatin remodeling as well as innate and acquired endocrine resistance. However, the role of LMTK3 in response to cytotoxic chemotherapy has not been investigated thus far. Using both 2D and 3D tissue culture models, we found that overexpression of LMTK3 decreased the sensitivity of breast cancer cell lines to cytotoxic (doxorubicin) treatment. In a mouse model we showed that ectopic overexpression of LMTK3 decreases the efficacy of doxorubicin in reducing tumor growth. Interestingly, breast cancer cells overexpressing LMTK3 delayed the generation of double strand breaks (DSBs) after exposure to doxorubicin, as measured by the formation of γH2AX foci. This effect was at least partly mediated by decreased activity of ataxia-telangiectasia mutated kinase (ATM) as indicated by its reduced phosphorylation levels. In addition, our RNA-seq analyses showed that doxorubicin differentially regulated the expression of over 700 genes depending on LMTK3 protein expression levels. Furthermore, these genes were found to promote DNA repair, cell viability and tumorigenesis processes / pathways in LMTK3-overexpressing MCF7 cells. In human cancers, immunohistochemistry staining of LMTK3 in pre- and post-chemotherapy breast tumor pairs from four separate clinical cohorts revealed a significant increase of LMTK3 following both doxorubicin and docetaxel based chemotherapy. In aggregate, our findings show for the first time a contribution of LMTK3 in cytotoxic drug resistance in breast cancer.

## Introduction

Lemur tyrosine kinase 3 (*LMTK3*; also known as *LMR3*, *TYKLM3*, *KIAA1883*) is a predicted dual-specificity protein kinase whose expression levels has been implicated in cancer cell invasion, endocrine resistance, poor prognosis and overall tumor progression in different types of malignancies [1–15]. Based on previous observations associating LMTK3 with tamoxifen resistance in breast cancer [1, 12], we set out to investigate a possible role of LMTK3 in breast cancer cells’ response to cytotoxic treatment in vitro and in vivo.

Adjuvant and neo-adjuvant chemotherapy still represent the backbone of systemic treatment for many cancers at both the early and advanced stages of the disease [16–18]. DNA-damaging agents are used in cancer chemotherapy, as DNA integrity is crucial for proper cellular function and proliferation [19, 20]. One of these drugs is doxorubicin, an anthracycline antibiotic, which intercalates into DNA, hindering topoisomerase II progression and leading to cytotoxicity mostly by inhibition of DNA replication and generation of double-strand breaks (DSBs). However, even though doxorubicin is a widely used drug in chemotherapy regimen for breast cancer patients [21], resistance (innate and/or acquired) is often observed [22]. Therefore, cellular mechanisms that relate to doxorubicin-related resistance need deciphering.

As mentioned above, inhibition of topoisomerase II by doxorubicin results in the formation of DNA DSBs [23, 24]. Ataxia-telangiectasia mutated kinase (ATM) is amongst the earliest kinases activated in the cellular response to DSBs [25, 26]. In addition, it has been shown that ATM kinase can be activated upon doxorubicin treatment [27–29]. This is achieved by the phosphorylation at Ser1981 leading to the dissociation of inactive ATM dimers and the generation of catalytically active monomers that later expose ATM kinase activity at the DSBs sites [30, 31].

Several studies have demonstrated that doxorubicin-induced DNA DSBs lead to the phosphorylation of histone H2AX at Ser139 via ATM [26, 32–35]. Histone H2AX is a substrate of several phosphatidylinositol 3-kinase-related kinases, such as ATM, ATM-and Rad3-related kinase (ATR) as well as DNA-dependent protein kinase. It has been described that phosphorylation of histone H2AX on Ser139 (γH2AX) represents the most frequent marker of the DNA damage response caused by DSBs [35–37], while the number of foci formation is proportional to the severity of the damage [38]. KRAB-associated protein 1 (KAP1) is another ATM substrate identified as a co-repressor of gene transcription. Doxorubicin-mediated ATM activation elicits phosphorylation of KAP1 at Ser824 and inhibits KAP1 sumoylation. Subsequently, ATM-dependent KAP1 Ser824 phosphorylation de-represses transcription of p21, Gadd45α, Bax, Puma and Noxa that promote cell cycle control and apoptosis in response to doxorubicin [27, 28].

In this study, we implicate LMTK3 in the protection of breast cancer cells from DNA DSBs as induced by doxorubicin. Using 2D monolayers as well as 3D-spheroids cultures we show that breast cancer cells stably overexpressing LMTK3 are more resistant to doxorubicin treatment when compared to the parental ones. Importantly, we underpin these results by using a breast cancer xenograft mouse model revealing that increased LMTK3 levels decrease the antitumor activity of doxorubicin.

Furthermore, we provide details underlying the effects of LMTK3 in doxorubicin induced DNA damage/repair processes. We demonstrate that MCF7 cells stably over-expressing LMTK3 delay H2AX Ser139 phosphorylation resulting in fewer γH2AX foci as compared to parental MCF7 cells. Our analysis also reveals that ectopic expression of LMTK3 delays DNA damage response by decreasing ATM phosphorylation.

To obtain information underlying the LMTK3-mediated doxorubicin resistance, we employed a whole genome transcriptomic analysis approach using RNA-sequencing (RNAseq). A plethora of bioinformatics analyses tools were implemented including DESeq2 (differential gene expression), the ingenuity pathway analysis (IPA; canonical pathways and disease/biological functions), the STRING database (protein–protein interaction analysis), and the Gene Ontology Enrichment Analysis (protein functions). As a result, we identified several genes and pathways that are differentially regulated by doxorubicin based on LMTK3 expression. In particular, we observed that doxorubicin treatment of LMTK3-overexpressing MCF7 cells shows a decrease in DNA damage sensing compared to parental MCF7 cells, while pathways involved in cell survival, cell viability and tumorigenesis are predominantly activated.

Finally, we examined the expression of LMTK3 in 148 pairs of primary breast cancer cases, before and after receiving chemotherapy. Interestingly, we detected a significant upregulation of LMTK3 protein levels, as assessed by immunohistochemistry, following doxorubicin and docetaxel based therapy. These results suggest that the ultimate resistance observed to these drugs could also be due to the aberrant over-expression of oncogenic LMTK3. In summary, our data illustrate a novel role and involvement of LMTK3 in doxorubicin resistance in breast cancer.

## Results

### Ectopic expression of LMTK3 decreases the cytotoxic efficacy of doxorubicin in vitro and in vivo

**Fig. 1:**
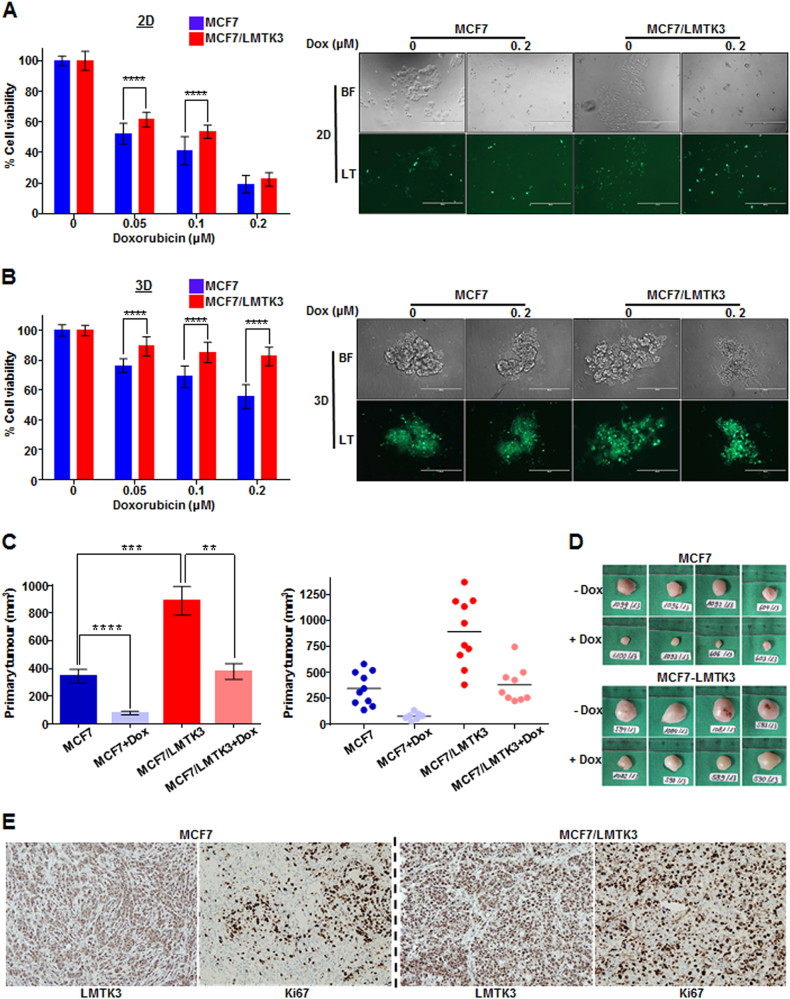
Over-expression of LMTK3 impedes the effectiveness of doxorubicin in vitro and in vivo. Following treatment with different concentrations of doxorubicin (0.05, 0.1, and 0.2 μM) for 72 h, the percentage (%) of cell viability, was assessed by CellTiter-Glo assay in MCF7 and MCF7/LMTK3 cells cultured in either (**a**) 2D (monolayers) or (**b**) 3D (spheroids). All error bars represent the mean ± the standard deviation (SD) from three independent experiments. **c** Quantification in primary tumor size between MCF7 and MCF7/LMTK3 xenografts with or without doxorubicin treatment at Day 28. (Left) The means and the error bars show the standard error of the mean (SEM) for ten MCF7-LMTK3, nine MCF7-LMTK3+Dox, ten MCF7 and seven MCF7+Dox –derived xenografts. (Right) Data are displayed as individual data points together with their corresponding median values. **d** Representative images of tumors at Day 28 are shown for the different groups. **(e)** Histological analysis of LMTK3 and Ki67 expression in representative tumor tissue sections of MCF7 and MCF7/LMTK3 tumors (no doxorubicin treatment). Original magnification, 100×. Scale bars, 100 μm. (**P ≤ 0.01; ***P ≤ 0.001; ****P ≤ 0.0001)

To study the physiological role of LMTK3 in response to chemotherapeutic drug treatment, MCF7 and MCF7/LMTK3 cells grown either in 2D or 3D cultures were treated with various doses of doxorubicin for different time points. The effects of LMTK3 overexpression on doxorubicin sensitivity, were assessed by cell viability and proliferation assays. MCF7/LMTK3 cells grown either in 2D (Fig. 1a and Supplementary Figure 1A, B) or 3D (Fig. 1b) were more resistant to doxorubicin-induced cytotoxicity, compared to the parental MCF7. Similarly, MDA-MB-231/LMTK3 cultured in 3D (Supplementary Figure 1C), displayed higher viability rates following doxorubicin treatment vs. the parental MDA-MB-231 cells, while the opposite results were observed when this cell line was cultured in 2D monolayer (Supplementary Figure 1D). As cells’ behavior is altered in 3D vs. 2D cultures, this result suggests that the actions of LMTK3 in MDA-MB-231 (triple negative cell line) may be influenced by various intrinsic and/or extrinsic factors as well as tissue architecture. The latter parameter is particularly important for breast cells as it has been shown that lactation stops when milk-secreting cells are cultured in 2D, while this feature is restored once cells are cultured in 3D [39–41]. In addition, as expected, treatment with doxorubicin in all cell lines was more active in 2D vs. 3D cultures [42–44]. Taken together, these data indicate that breast cancer cells overexpressing LMTK3 are less sensitive (or more resistant) to doxorubicin treatment, compared to their respective parental cell lines expressing basal levels of LMTK3.

To evaluate the impact of LMTK3 in the antitumor efficacy of doxorubicin in vivo, female athymic nude mice in a xenograft tumor model. MCF7 and MCF7/LMTK3 cells were subcutaneously injected into the right flank of mice. On day 10, animals were randomized in different groups and doxorubicin was administered intravenously to the respective groups on the same day and one week later (Group 1: MCF-7; Group 2: MCF7+Dox; Group 3: MCF7/LMTK3; Group 4: MCF7/LMTK3+Dox). Tumor volume was determined in vivo by external caliper on the indicated time points. On day 28, mice were sacrificed and tumors were dissected and photographed. As anticipated, LMTK3 overexpression increased tumor growth, as previously observed in an in vivo study using T47D and T47D/LMTK3 breast cancer cell lines [13] further supporting its oncogenic role. In addition, doxorubicin treatment significantly inhibited tumor growth in both the MCF7 and the MCF7/LMTK3 xenograft mice. However, in the presence of doxorubicin, the tumor growth inhibition was reduced in the MCF7/LMTK3 group (*P* = 0.0022) compared to the MCF7 one (*P* < 0.0001) (Figs. 1c, d and Supplementary Figure 2), data that were in line with our in vitro results above. Finally, LMTK3 overexpression was also associated with increased tumor cell proliferation as assessed by Ki-67 immunohistochemistry analysis. As shown in the representative images (Fig. 1e), abundant Ki67-positive nuclei were observed mostly in the tumors of the MCF7/LMTK3 mice compared to the MCF7 ones.

### LMTK3 over-expression delays doxorubicin-induced DNA DSBs

Exposure to doxorubicin generates DSBs, leading to the phosphorylation of histone H2AX on Ser139. The γH2AX formation is a major, rapid and sensitive marker of DSB induction [45]. Hence, to further explore the doxorubicin-resistant phenotype of LMTK3 over-expressing cells, we examined the time dependency of γH2AX foci formation following doxorubicin treatment by immunofluorescence

**Fig. 2:**
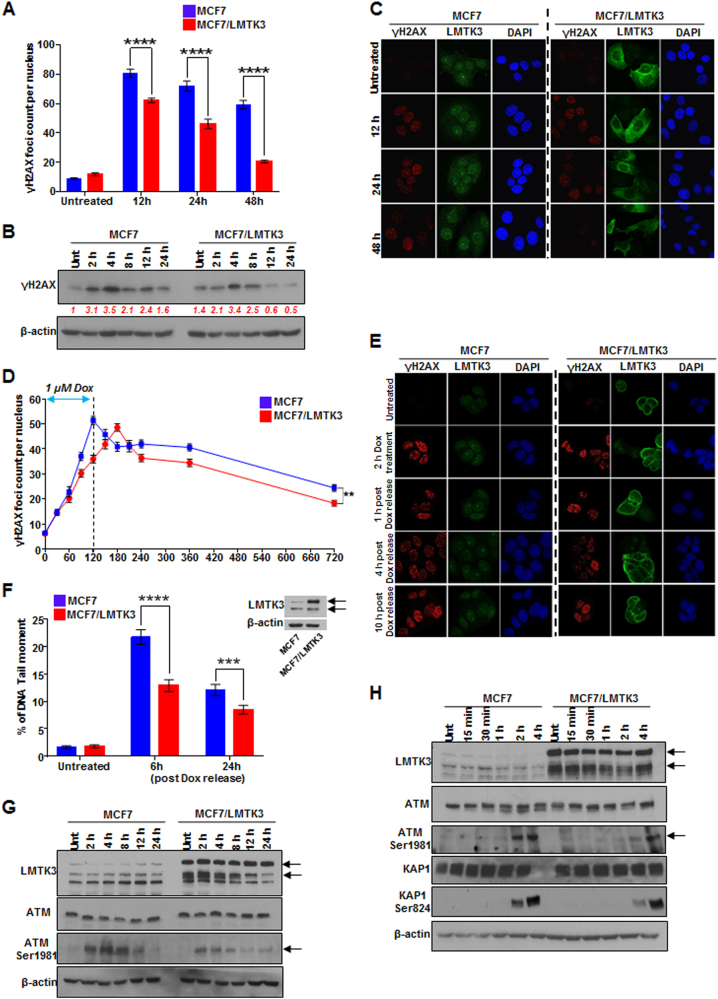
Overexpression of LMTK3 delays doxorubicin-induced DNA DSBs and affects the phosphorylation of ATM. **a** MCF7 and MCF7/LMTK3 cells were treated with 0.4 µM of doxorubicin and 12, 24, and 48 h later, quantitative analysis of γH2AX foci was performed. All error bars represent the mean ± SEM (50 cells were analyzed per each time point; ****P ≤ 0.0001). **b** Western blot analysis for endogenous γH2AX levels in MCF7 and MCF7/LMTK3 cells following doxorubicin treatment for different time points. β-actin was used as loading control. **c** MCF7 and MCF7/LMTK3 cells were fixed and subjected to confocal immunofluorescence analysis. Representative confocal microscopy images for γH2AX and LMTK3 are shown for different time points. **d** MCF7 and MCF7-LMTK3 cells were treated with 1 µM of doxorubicin for up to 2 h. Following, the media was removed allowing cells to grow in fresh complete media for another 10 h. Quantitative analysis of γH2AX foci was performed. All error bars represent the mean ± SEM (50 cells were analysed per each time point; **P ≤ 0.01). **e** MCF7 and MCF7/LMTK3 cells were fixed and subjected to confocal immunofluorescence analysis. Representative confocal microscopy images for γH2AX are shown for different time points following doxorubicin release. **f** Doxorubicin-induced DNA damage measured by neutral comet assay. MCF7 and MCF7-LMTK3 cells were treated with 1 µM of doxorubicin for up to 2 h. Following, the media was removed allowing cells to grow in fresh complete media for another 6 and 24 h. Cells were analyzed by fluorescence microscopy; The tail moment was quantified using the ImageJ software with OpenComet plug-in. All error bars represent the mean ± SEM (51 cells were analysed per each time point; ***P ≤ 0.001; ****P ≤ 0.0001). A representative western blot of LMTK3 expression in MCF7 and MCF7/LMTK3 cell lines is shown. **g** MCF7 and MCF7-LMTK3 cells were treated with 1 µM of doxorubicin for 2, 4, 8, 16, and 24 h. Western blot analysis was performed using the respective antibodies. β-actin was used as loading control. Representative results and quantification of protein bands using ImageJ software are shown for two separate blots. **h** MCF7 and MCF7-LMTK3 cells were treated with 1 µM of doxorubicin for 15 min, 30 min, 1, 2, and 4 h. Western blot analysis was performed using the respective antibodies. β-actin was used as loading control. Representative results and quantification of protein bands using ImageJ software are shown for two separate blots

MCF7 and MCF7/LMTK3 cells were exposed to 0.4 μM of doxorubicin and incubated for 12, 24, and 48 h. Untreated cells contained few γH2AX foci per cell (8.8 ± 1.21 foci in MCF7, 11.8 ± 2.01 in MCF7/LMTK3; Fig. 2a) with no statistically significant difference between the two cell lines. As expected, H2AX phosphorylation was increased in a time-dependent manner upon doxorubicin exposure compared to the respective untreated cells. Interestingly, γH2AX accumulation was significantly lesser in MCF7/LMTK3 cells as compared to the MCF7 ones, at various time points during doxorubicin exposure as demonstrated by western blot and immunofluorescence analyses (Figs. 2b, c and Supplementary Figure 3). These results suggest that LMTK3 may attenuate DNA damage by playing a role in the formation of DSBs as determined by the phosphorylation of the histone variant H2AX.

We then sought to determine whether the rapid resolution in γH2AX foci formation at 12, 24 or 48 h was due to delayed resolution of damage or increased induction of DNA repair, foci were quantified at earlier time points. Following 2 h of doxorubicin treatment (1 µM), to induce DNA damage, the drug was removed and cells continued being cultured in fresh complete media for additional 10 h. The γH2AX kinetics followed a biphasic curve; first, a rapid phase of foci formation followed by an exponential γH2AX foci decay phase. Although the initial number of foci was nearly identical in both MCF7 and MCF7/LMTK3 cells at 30 min, indicating similar levels of induced DNA damage, the number of foci present 120 min after doxorubicin treatment was clearly lower in MCF7-LMTK3 cells (35.98 ± 1.57) compared to MCF7 cells (51.42 ± 1.64) (Fig. 2d). In addition, LMTK3 over-expression delayed this response as the foci increased gradually and culminated at 180 min (48.58 ± 1.48). However, despite the time shift (~60 min) in the peaks observed in MCF7 and MCF7/LMTK3, there was no statistically significant difference between the peaks of the two cell lines (MCF7: 51.42 ± 1.64 vs. MCF7/LMTK3: 48.58 ± 1.48). Interestingly, although the decay curve appears similar, analysis of the time course for the disappearance of foci in MCF7 and MCF7/LMTK3 cells after exposure to doxorubicin showed that the foci in MCF7/LMTK3 cells disappeared more rapidly, compared with that observed in MCF7 cells. In the case of MCF7 cells, 10 h post γH2AX foci peak formation, the number of foci reduced ~52% (from 51.42 ± 1.64 to 24.44 ± 1.35). In the case of MCF7/LMTK3 cells, 9 h after γH2AX foci peak formation the number of foci reduced ~62% (from 48.58 ± 1.48 to 18.32 ± 1.15) (Figs. 2d, e and Supplementary Table 1).

Finally, we used comet assays (single-cell gel electrophoresis) that can detect a variety of DNA damage (single or DSBs and DNA crosslinks) as well as for monitoring DNA repair [46, 47]. Cells were treated with 1 µM of doxorubicin for 2 h to induce DNA breaks. Following, the media was replaced with fresh one allowing cells to grow for another 6 and 24 h. Doxorubicin caused the “comet” migration pattern characteristics, while quantitative analysis showed that overexpression of LMTK3 (MCF7/LMTK3) led to significantly lower DNA tail moments when compared to MCF7 cells (12.89 ± 1.10 vs. 21.71 ± 1.35 for 6 h (*P* < 0.0001) and 8.49 ± 0.80 vs. 12.10 ± 1.00 for 24 h (*P* = 0.0043)) (Fig. 2f and Supplementary Table 2), which is consistent with the reduced γH2AX foci observed in our previous experiments. Collectively, these data demonstrate that LMTK3 has a role in delaying doxorubicin-induced DNA damage and is potentially involved in the DNA repair mechanisms.

### LMTK3 impedes γH2AX foci formation by influencing the activity of ataxia-telangiectasia mutated kinase (ATM)

Next, the effects of LMTK3 overexpression on ATM were evaluated. MCF7 and MCF7/LMTK3 cells were exposed to doxorubicin for up to 24 h and ATM phosphorylation status was examined. Consistent with previous reports [27, 28], ATM phosphorylation at Ser1981 in MCF7 cells, peaked within 2–4 h of exposure to doxorubicin and was gradually decreased over time before reaching its basal levels 16 h later. In contrast, MCF7/LMTK3 cells displayed weaker total ATM phosphorylation compared to MCF7, while the signal appeared highest after 4 h of doxorubicin treatment and decayed faster (~8 h of doxorubicin treatment) (Fig. 2g). Since ATM is involved in the early DNA damage response, cells were treated and harvested at shorter time points. Similarly, phosphorylation of ATM at Ser1981 in MCF7/LMTK3 cells was lesser in the initial 2 h of doxorubicin treatment compared to MCF7 (Fig. 2h). Finally, we also checked for the phosphorylation of KAP1, which represents a novel ATM substrate that is involved in DNA damage [28]. In MCF7/LMTK3 cells, phosphorylation of KAP1 at Ser824, after 2 h of doxorubicin treatment, was less than half as compared to MCF7 cells, following a similar pattern with the activation of ATM (Fig. 2h). Taken together, these data imply that over-expression of LMTK3 may delay the sensing to DNA damage through the ATM signaling pathway.

### Doxorubicin differentially regulates global transcriptome changes based on LMTK3 status

To gain additional insights into the molecular mechanisms and pathways involved in LMTK3 mediated chemo-resistance, we performed transcriptome profiling by RNA-seq in MCF7 and MCF7/LMTK3 cells treated with 1 μM of doxorubicin for 24 h or vehicle only (DMSO; control). Principal component analysis (PCA) and sample clustering analysis of the RNA-Seq data showed a high degree of similarity between replicate samples (Supplementary Figure 4A and B). Interestingly, the PCA plot revealed a greater doxorubicin-mediated transcriptomic variation in MCF7/LMTK3 cells in comparison to MCF7 cells (Supplementary Figure A). This suggests a potential interaction between LMTK3 and doxorubicin pathways in MCF7 cells.

**Fig. 3:**
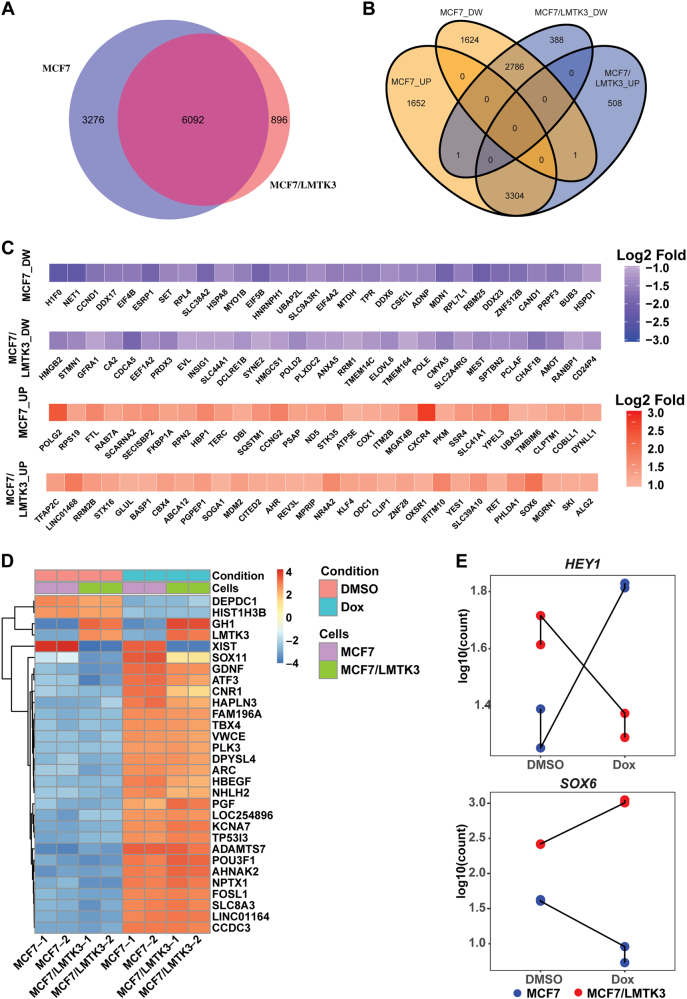
Global transcriptomic alterations induced by doxorubicin in MCF7 and MCF7/LMTK3 cells. **a** Venn diagram showing a high degree of overlap as well as differences in the transcripts significantly regulated by doxorubicin in MCF7 and MCF7/LMTK3 cells (*P*_adj_ < 0.05 and Log2 fold difference of ≥|1|). **b** Venn diagram comparing numbers of transcripts significantly up (UP) or down-regulated (DW) in each dataset. **c** Heatmaps showing top-30 (ordered based on *P*_adj_ value) exclusively regulated transcripts upon doxorubicin treatment of MCF7 and MCF7/LMTK3 cells. Downregulated transcripts (DW) are shown in the upper panel, while upregulated transcripts (UP) are shown in the lower panel. **d** Heatmap showing amounts by which the read counts of the top-30 (ordered based on *P*_adj_ value) commonly regulated genes deviates from the gene’s average across all the samples. **e** Count plot comparing the changes in the RNA-seq read counts of *HEY1* and *SOX6* between MCF7 and MCF7/LMTK3 cells upon treatment with DMSO or 1 μM doxorubicin

We next employed the differential gene expression analysis using the DESeq2 pipeline to identify genes induced by doxorubicin in MCF7 and MCF7/LMTK3 cells. In summary, a total of 9368 and 6988 transcripts were detected to be significantly regulated by doxorubicin in MCF7 and MCF7/LMTK3 respectively (Fig. 3a and Supplementary excel files 1 & 2). Approximately, 25% of doxorubicin regulated transcripts were unique to either MCF7 or MCF7/LMTK3, hence, providing further evidence for differential gene regulation by doxorubicin based on expression levels of LMTK3. To gain additional insights into similarities and differences in doxorubicin effect, we plotted (Venn diagram) genes identified to be regulated by doxorubicin either in MCF7 or MCF7/LMTK3 cells at the false discovery rate *P*_adj_ < 0.05 and Log2 fold difference of ≥|1|. We found that doxorubicin treatment upregulated 4957 and 3813 transcripts and downregulated 4411 and 3175 transcripts in MCF7 and MCF7/LMTK3 cells respectively (Fig. 3b and Supplementary excel files 1 & 2). Of these transcripts, 3304 and 2786 genes were commonly regulated, and 3276 and 896 were uniquely regulated by doxorubicin in MCF7 and MCF7/LMTK3 respectively. Moreover, we also generated heatmaps displaying the amounts by which the read-counts of the top-30 genes identified as uniquely or commonly regulated by doxorubicin across MCF7 and MCF7/LMTK3 cells deviate from the genes’ average across all the samples (Figs. 3c, d and Supplementary excel file 3). To validate our results, we performed qRT-PCR analysis in 14 randomly picked genes, including SOX6 and HEY1 that were predicted to be differentially expressed. We found a strong validation (14/14) of our RNAseq and DESeq2 analysis in a separate cohort of samples (Supplementary Tables 3 & 4). We did observe a difference in the fold-difference as calculated by DESEq2 and the qRT-PCR technique. However, this is expected as fold-difference predicted by DESEq2 accounts for reads aligning to the entire gene, while qRT-PCR is less sensitive as it is based on a defined region (amplified by primer set) within the gene.

Our Venn diagram also pointed to an intriguing result where doxorubicin differentially regulated the expression of SOX6 and HEY1 transcription factors in MCF7 and MCF7/LMTK3 cells (Fig. 3e). In particular, doxorubicin suppressed the expression of SOX6 in MCF7 cells (~ 6-fold), whereas it increased it in MCF7/LMTK3 cells (~4-fold). In contrast, doxorubicin potentiated the expression of HEY1 by ~3 × fold in MCF7 cells and suppressed it by ~2 × fold in MCF7/LMTK3 (Fig. 3e).

**Fig. 4:**
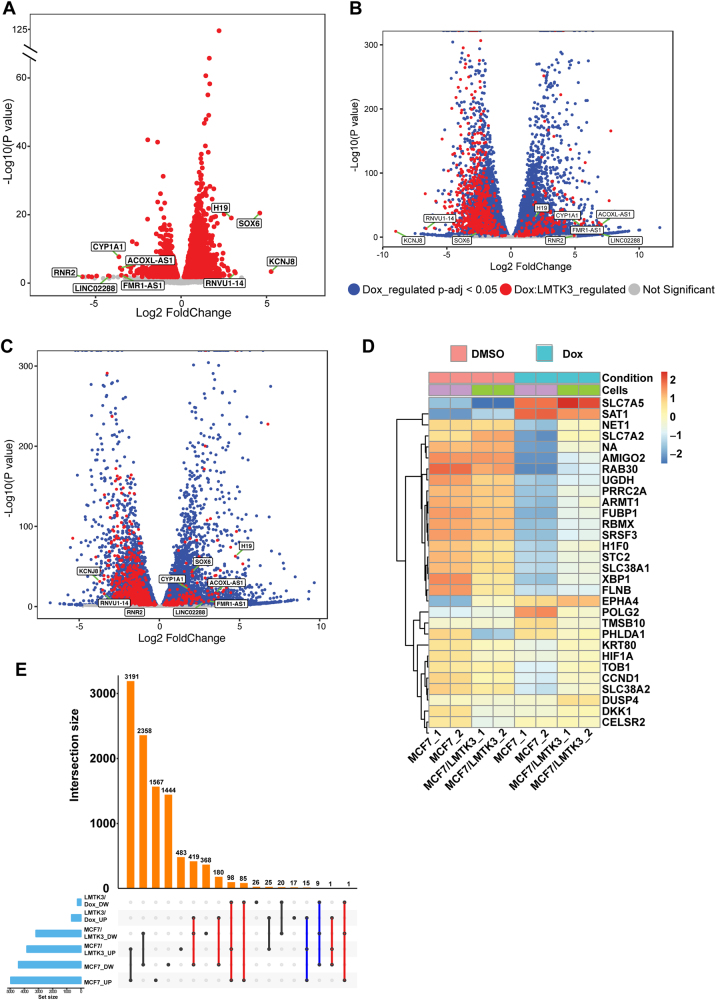
Differences in doxorubicin induced global transcriptomic changes based on LMTK3 expression levels. **a** Volcano plot showing the Log2 fold change of genes that respond differently to the doxorubicin treatment in MCF7 and MCF7/LMTK3 cells (Dox:LMTK3). The Log10 of *P* value, for significance in fold change, is plotted on the *y*-axis. **b** Volcano plot showing the Log2 fold change of doxorubicin regulated genes in MCF7 cells. Genes are colored based on their mode of regulation. Genes identified to respond differently to doxorubicin treatment in MCF7 and MCF7/LMTK3 cells are colored red (Dox:LMTK3). In addition, genes regulated commonly by doxorubicin treatment in both cell lines are colored blue. Genes were colored grey if their fold change were not significantly different compared to DMSO. **c** Volcano plot showing the Log2 fold change of doxorubicin regulated genes in MCF7/LMTK3 cells. Genes are colored based on their mode of regulation as described above. Top Dox:LMTK3 genes (based on Log2 fold chage) are labelled on all the volcano plots. **d** Heatmap showing amounts by which the read counts of the top-30 Dox:LMTK3 genes (ordered based on *P*_adj_ value) deviates from the gene’s average across all the samples. **e** The UpSet plot showing common and unique doxorubicin regulated genes significantly up (UP) or down-regulated (DW) in each dataset. The red lines indicate antagonistic regulation of same genes by doxorubicin across MCF7 and MCF7/LMTK3 cells. The blue lines indicate additive regulation of genes by doxorubicin across MCF7 and MCF7/LMTK3 cells

To further inquire if there were additional genes that differentially responded to doxorubicin treatment between MCF7 and MCF7/LMTK3 cells (from now on labelled as: Dox:LMTK3 genes), we re-analyzed the RNA-Seq data using the interaction model provided by DESeq2. This model tests for genes that respond differently to doxorubicin treatment across MCF7 and MCF7/LMTK3 by controlling for differences between cell lines due to LMTK3 overexpression and doxorubicin treatment effect on MCF7 cells. The model identified that 896 genes responded differently (at the *P*_adj_ < 0.05 and Log2 fold difference of ≥1 or ≤−1) to doxorubicin treatment in MCF7 and MCF7/LMTK3 cells (Fig. 4a and Supplementary excel file 4). To further distinguish these genes from those regulated commonly by doxorubicin in both cell lines, we plotted the Log2 fold expression changes vs. Log10 *P*-values and cataloged genes according to their mode of regulation (Figs. 4b, c). Moreover, we generated heatmaps displaying the amounts by which the read-count of the top-30 Dox:LMTK3 genes deviate from the genes’ average across all the samples (Fig. 4d).

Next, to understand how LMTK3 overexpression affects doxorubicin-mediated gene expression we compared and contrasted expression of Dox:LMTK3 genes across all the samples using the intersection UpSet plot (Fig. 4e). This revealed an interesting antagonistic regulation of 784 genes by doxorubicin in MCF7 and MCF7/LMTK3 cells (Fig. 4e; red lines). We found that when compared to doxorubicin-treated MCF7 cells, doxorubicin-treatment in MCF7/LMTK3 cells had a significantly lower (*P*_adj_ ≤ 0.05 and Log2 fold ≤ −1) upregulation and downregulation of 184 and 600 transcripts, respectively. Furthermore, the plot revealed a small synergistic effect between LMTK3 overexpression and doxorubicin-mediated regulation of 26 genes (Fig. 4e; blue lines). In comparison to doxorubicin-treated MCF7, we found a significantly higher (*P*_adj_ ≤ 0.05 and Log2 fold ≥ 1) downregulation and upregulation of 9 and 15 transcripts respectively in MCF7/LMTK3 cells.

### Functional analysis of doxorubicin mediated differential gene expression

**Fig. 5:**
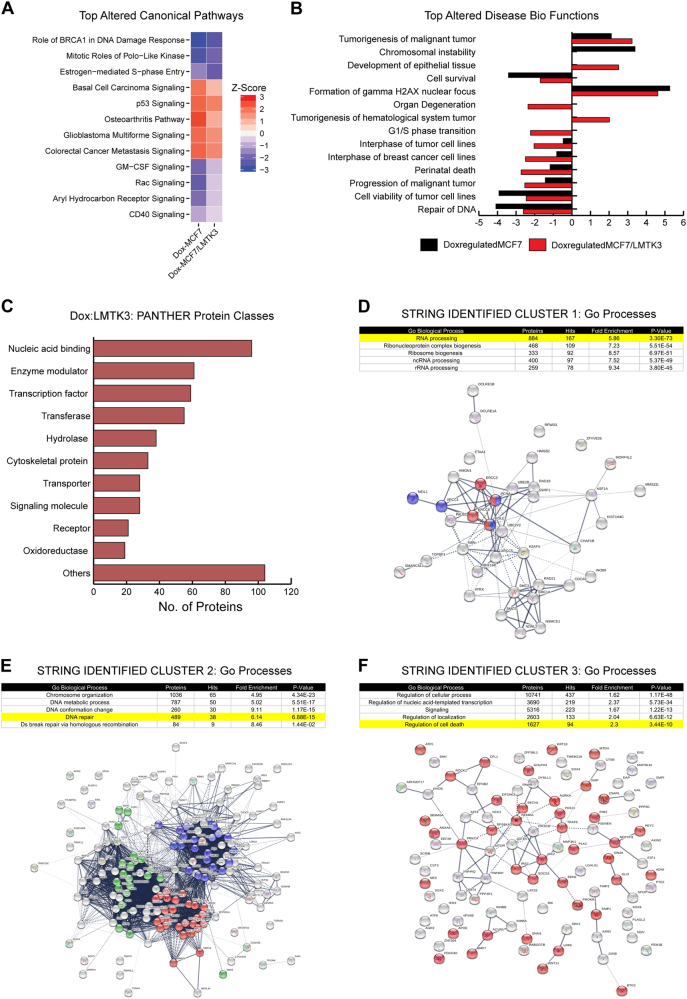
Functional analysis of doxorubicin-LMTK3 mediated differential gene expression. **a** Heatmaps comparing *Z* scores of canonical pathways significantly enriched for doxorubicin regulated genes identified from doxorubicin/DMSO treated MCF7 and MCF7/LMTK3 cells. The significant *P*-values were calculated by Fisher’s exact test. The activation or inhibition of the canonical pathways and disease bio functions is given by *Z* score. A *Z* score of ≥2 is considered as significant activation and a *Z*-value of ≤−2 is considered as significant inhibition. The *Z* score between (0, 2) or (−2, 0) represents trend towards activation or inhibition, respectively. **b** A bar graph comparing *Z* scores of disease biological functions enriched for doxorubicin regulated genes identified from doxorubicin/DMSO treated MCF7 and MCF7/LMTK3 cells. **c** Functional classification of Dox:LMTK3 genes identified using PANTHER classification system. **d–f** GO pathways analysis of the protein-protein interaction clusters identified in Dox:LMTK3 genes using fast-greedy algorithm provided with STRING database. The STRING network analysis was then performed on gene products involved in RNA processing (**d**: Cluster 1), DNA repair (**e**: Cluster 2), and regulation of cell death (**f**: Cluster 3). The blue, red and green color in STRING network of RNA processing represents proteins involved in splicesome, ribosome and ribosome biogenesis respectively. The red and blue color in STRING network of DNA repair represents proteins involved in Nucleotide Excision Repair and Base Excision Repair. The red color in STRING network of regulation of cell death represents proteins involved in downregulation of apoptotic pathways. For all the STRING networks, the strength of the black line indicates strength of the data support for a given protein-protein association

One of the most interesting RNAseq analysis observations was related to functional pathways that were differently altered in doxorubicin-treated MCF7 and MCF7/LMTK3 cells. We employed the IPA algorithm, which compares observed gene expression changes in our dataset with what is known from the current literature, to predict alterations in canonical pathways, biological functions, and upstream regulatory molecules. Using IPA, we found several pathways including estrogen-mediated S-phase entry, basal cell carcinoma signaling, osteoarthritis pathway, and P53 signaling that were either inhibited or had a trend towards inhibition (based on *Z* score) in doxorubicin-treated MCF7/LMTK3 cells compared to doxorubicin-treated MCF7 cells. In contrast, pathways such as mitotic roles of Polo-like kinase, Rac signaling, aryl hydrocarbon receptor signaling, GM-CSF signaling, and CD40 signaling were activated or had a trend towards activation in doxorubicin-treated MCF7/LMTK3 cells compared to MCF7 (Fig. 5a and Supplementary excel file 5**)**. The IPA also revealed a significant decrease in doxorubicin-mediated inhibition of several biological functions including cell survival, cell viability of tumor cells and DNA repair, as well as, a significant decrease in doxorubicin-mediated activation of biological functions such as cell death of tumor cells, the formation of γH2AX and chromosomal instability in doxorubicin-treated MCF7/LMTK3 cells compared to doxorubicin-treated MCF7 cells (Fig. 5b and Supplementary excel file 6).

Furthermore, using IPA we found five upstream regulators including ESR1 (*P* = 3.87 × 10^−7^), NUPR1 (*P* = 2.95 × 10^−4^), Rb (*P* = 0.00595), BRD4 (*P* = 0.0123), and miR-16-5p (*P* = 0.0207) whose downstream target genes responded differently to doxorubicin treatment of MCF7 **(**Supplementary excel file 7**)** and MCF/LMTK3 **(**Supplementary excel file 8**)** cells. These upstream regulators may offer an explanation for why doxorubicin had a different effect on gene expression in MCF7 and MCF7/LMTK3 cells (Supplementary Figure 5). Furthermore, we also performed an upstream regulator analysis with Dox:LMTK3 genes (Supplementary excel file 9).

Clustering the Dox:LMTK3 genes based on their protein functions can offer a distinctive insight into the how cellular activities differ in doxorubicin-treated MCF7 and MCF7/LMTK3 cells. Therefore, we first identified the PANTHER protein class of Dox:LMTK3 genes (Fig. 5c, Supplementary excel file 10). Next, we found three clusters of functionally interacting proteins using the fast-greedy algorithm in R. We then performed three separate GO enrichment analyses for proteins enriched in each cluster and finally mapped STRING protein networks for Dox:LMTK3 proteins enriched in interesting pathways (Figs. 5d, e, f). Our analyses revealed that a vast majority of Dox:LMTK3 genes synthesized nucleic acid binding proteins, transcription factors and enzyme modulators. Furthermore, the clustering and GO enrichment analyses identified proteins enriched in pathways known to be associated with doxorubicin treatment such as transcription regulation [48], double-stranded break repair, DNA repair [49] and cellular apoptosis [50] (Figs. 5d, e, f and Supplementary excel files 11–13). Intriguingly, the analysis also identified pathways such as mRNA processing, cell-adhesion, and chromatin remodeling that were not expected to be differentially regulated by doxorubicin in MCF7 and MCF7/LMTK3 cells. Taken together, the RNAseq data revealed dysregulation within the canonical pathways, biological functions, protein interaction and upstream regulators that offer additional insight into the observed doxorubicin resistance in MCF7/LMTK3 cells.

### Breast cancer chemotherapy affects LMTK3 expression levels

**Fig. 6:**
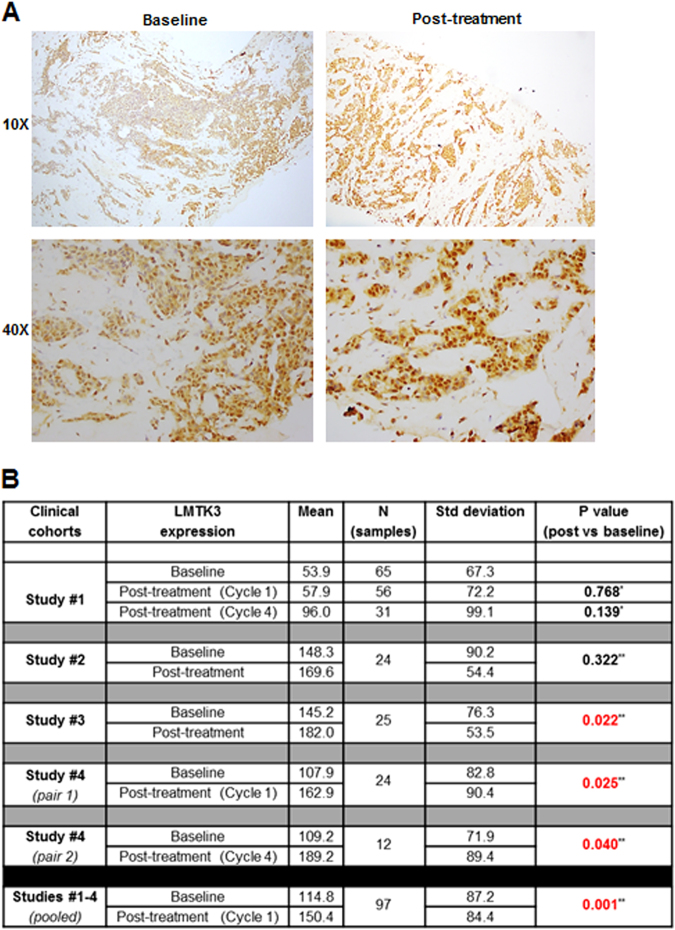
LMTK3 expression in pre and post-chemotherapy primary breast tumors. **a** Representative histological images of LMTK3 expression in matched pre- and post-chemotherapy biopsies (docetaxel and doxorubicin) of a breast cancer patient from study/cohort #3. **b** Scoring of nuclear LMTK3 expression levels in individual and pooled breast cancer cohorts receiving chemotherapy treatment

Lastly, we examined the expression levels of LMTK3 in 148 breast cancer patients from 4 individual studies, pre and post-chemotherapy treatment. Representative immunohistochemistry staining of LMTK3 expression is shown in Fig. 6a. When data from all four studies were pooled, a significant increase in LMTK3 expression with chemotherapy was observed (mean LMTK3 expression 150.2 ± 84.4 vs. 114.8 ± 87.2 for post cycle 1 vs. pre-treatment; *P* = 0.001) (Fig. 6b). Interestingly, when data from studies 1 and 4, where 3 serial pre and post-treatment tumor biopsies were obtained from each patient, were pooled, progressive increase in tumor LMTK3 expression was noted with increasing number of chemotherapy cycles (mean LMTK3 expression 67.6 ± 74.9, 94.6 ± 96.0, 135.1 ± 108.1 for pre, post cycle 1, and post cycle 4-chemotherapy, *P* = 0.006 for post cycle 1 vs. baseline, P = 0.020 for post cycle 4 vs. baseline).

## Discussion

We and others have demonstrated that LMTK3 expression is significantly elevated in high-grade breast tumors and is associated with poor survival rates in different breast cancer cohorts [1, 5]. Considering its involvement in endocrine resistance further investigation into its potential role as a therapeutic target is required.

Neoadjuvant and adjuvant chemotherapy in breast cancer has improved overall survival [51, 52]. However, resistance to cytotoxic chemotherapy is the main cause of therapeutic failure and death in women with breast cancer [53, 54]. Bearing in mind the increasing interest of 3D cell culture systems in cancer research, we sought to investigate and compare how LMTK3 affects sensitivity to cytotoxic treatment using the 3D models as well as the traditional 2D monolayers. Our 2D and 3D in vitro assays revealed that LMTK3 over-expression results in a significant increase in cell viability / proliferation in the presence of doxorubicin. Despite the high doxorubicin-induced cell death observed in both parental and LMTK3 overexpressed cell lines, there was a clear indication that LMTK3 appears to contribute resistance to this drug. More importantly, the results were recapitulated in vivo by examining the antitumor efficacy of doxorubicin in a breast cancer xenograft tumor model. Together, these findings describe a previously undescribed role of LMTK3 in doxorubicin resistance.

Formation of γH2AX foci starts within seconds after induction of DSBs, although they may also be present in small numbers even in the absence of exogenous damage. This can be due to DNA damage that can occur during replication, viral infection, senescence, carcinogenic adducts, exposure to reactive oxygen species and other cellular processes [55]. In an attempt to investigate the potential role of LMTK3 in doxorubicin-induced DNA DSBs, the kinetics of H2AX phosphorylation at Ser139 (γH2AX) foci formation and DNA breaks were measured. Interestingly, following exposure to doxorubicin, γH2AX foci formation were initially delayed while the % of DNA in the tail, reflecting the number of DNA breaks [56], was significantly lower in cells over-expressing LMTK3 following exposure to doxorubicin, suggesting that LMTK3 is implicated in the DNA damage response process.

Following, in order to elucidate the underlying mechanistic role of LMTK3 in doxorubicin-induced DNA damage and H2AX phosphorylation, the upstream ATM kinase was examined. Consistent with previous reports [27–29], exposure to doxorubicin induced phosphorylation of ATM at Ser1981 in MCF7 cells. However, phosphorylation of ATM at Ser1981 was significantly impaired in cells overexpressing LMTK3, which can explain the subsequent downstream reduction of H2AX Ser139 phosphorylation. Further investigation in the exact mechanism via which LMTK3 hinders ATM phosphorylation in response to DNA damage will provide us with essential information about the role of LMTK3 in this signaling pathway.

Phosphorylation of KAP1 at Ser824 by ATM, exclusively at DNA lesions, leads to chromatin relaxation and stimulate cells’ sensitivity to DSB-inducing agents [57, 58]. LMTK3 overexpression decreased KAP1 phosphorylation at Ser824, at least partly through the interaction with PP1α, as we have recently described [14]. Our new findings suggest that the LMTK3-induced decreased KAP1 phosphorylation can also occur (direct or indirectly) as a result of reduced ATM activity. Taken together, LMTK3 affects the phosphorylation of KAP1 by keeping it in an active co-repressor state that delays the induction of DNA damage and the de-repression of LMTK3-bound tumor suppressor-like genes (i.e., cell cycle control and apoptosis).

Interestingly, our RNA-seq data revealed that doxorubicin differentially regulated the expression of 784 genes in MCF7 and MCF7/LMTK3 cells. Of these 784 genes, SOX6 and HEY1 had a dramatic change in response to doxorubicin treatment in MCF7 and MCF7/LMTK3 cells. It is worth remarking that SOX6 [59] and HEY1 [60] are transcription factors associated with esophageal squamous cell carcinoma and breast cancer respectively, even though their roles in breast cancer and chemotherapy have not been thoroughly defined. Therefore, it would be interesting to further study their impact in breast cancer development as well as in chemotherapy resistance. The RNAseq results can be further used to generate new hypotheses to uncover additional mechanisms underlying LMTK3 mediated resistance to chemotherapy. For example, our analysis found that in MCF7/LMTK3 cells, the overexpression of LMTK3 affected a large number of Estrogen receptor alpha (ESR1) target genes (Supplementary excel files 5 and 6). Importantly, a significant fraction of those genes was changed in a direction that suggests decrease in doxorubicin-mediated inhibition of the aryl hydrocarbon receptor signaling, a pathway known to drive breast cancer progression. Hence, it would be interesting to validate whether the activation of aryl hydrocarbon receptor signaling and ESR1 would be sufficient to induce resistance to doxorubicin. In aggregate, our RNAseq data have revealed dysregulation in several canonical pathways and upstream targets that can offer additional insights into the observed LMTK3-mediated doxorubicin resistance in MCF7 cells.

Finally, in a clinical setting, after analysing pre- and post-chemotherapy samples from breast cancer patients, we detected a positive correlation between increased number of chemotherapy cycles and LMTK3 protein levels. These data were in accordance with our previous observations demonstrating a tendency of LMTK3 up-regulation with ongoing neo-adjuvant chemotherapy (doxorubicin / docetaxel) [5]. This intriguing result, in combination with the already established oncogenic role of LMTK3, requires further investigation as it suggests that prolonged chemotherapy might actually have adverse effects for certain patients. In addition, it will be interesting to elucidate the mechanism and feedback-loop signaling pathways via which doxorubicin, with or without docetaxel, promotes the over-expression of LMTK3, as we have seen in the matched clinical samples.

In summary, this is the first study to establish a new function of LMTK3 involvement in doxorubicin resistance. Although there are still many questions to address, our current data suggest a multifaceted role of LMTK3 in response to doxorubicin-induced cytotoxicity.

## Materials and methods

### Cell lines and reagents

MCF7 and MDA-MB-231 breast cancer cell lines (ATCC; STR authenticated) were maintained in Dulbecco’s Modified Eagle’s Medium (DMEM, Sigma-Aldrich) supplemented with 100 Units/ml penicillin, 100 µg/ml streptomycin, 2 mM L-glutamine (PSG, Sigma-Aldrich) and 10% heat inactivated fetal bovine serum (FBS, First Link). MCF7/LMTK3 and MDA-MB-231/LMTK3, stably over-expressing LMTK3, were cultured in DMEM supplemented with 10% FCS, G418 (500 μg/ml; Invitrogen) and 1% PSG. All cells were incubated at 37 °C in humidified 5% CO_2_, and were frequently tested for mycoplasma contamination. Antibodies used: LMTK3 (sc-100418, Santa Cruz Biotechnology); LMTK3 (H00114783-M02, Abnova); β-actin (ab627, Abcam); phospho-histone H2A.X Ser139 (2577, Cell Signaling Technology); phospho-histone H2A.X Ser139 (9718, Cell Signaling Technology); ATM (sc-23921, Santa Cruz Biotechnology); phospho-ATM Ser1981 (4526, Cell Signaling Technology); KAP1 (ab10484, Abcam); phospho-KAP1 Ser824 (ab70369, Abcam). Doxorubicin hydrochloride (D1515) was purchased from Sigma-Aldrich.

### 3D spheroid cultures

3D spheroids were generated from different breast cancer cell lines (MCF7, MCF7/LMTK3, MDA-MB-231 and MDA-MB-231/LMTK3) using 96-well round-bottom Ultra Low Attachment (ULA) plates (Corning®, New York, USA) that feature a hydrogel layer, which inhibits cellular attachment. Briefly, using ULA plates, 2.5 × 10^3^ cells/well were seeded in 30 µl of complete medium. Immediately after seeding, the plates were centrifuged at 300 × g for 3 min, and maintained at 37 °C in humidified 5% CO^2^ for 72 h. Following spheroids formation, 3D cultures were treated and analyzed accordingly, as described below.

### Cells labelling

Cells were collected, counted and resuspended to a final concentration of 1 × 10^6^ in serum free medium. The green fluorescent, lipophilic carbocyanine SP-DiOC18(3) (3,3′-dioctadecyl-5,5′-di(4-sulfophenyl) Oxacarbocyanine, Sodium Salt) (thermofisher) was added to the cell suspension to a final concentration of 1 µM. After 2 h at 37 °C in humidified 5% CO^2^, cells were centrifuged at 300×g for 5 min and cell pellets were washed twice with 1 ml of PBS. Labelled cells (excitation 475–495 nm, and emission 520–560 nm) were detected using the EVOS Cell Imaging Systems (thermofisher). Stained cells were used for further analysis.

### Cell viability / proliferation assays

Cell viability was assessed using the CellTiter-Glo assay (Promega) following the manufacturer’s instructions and the sulphorhodamine B growth assay, as previously described [61]. Briefly, 2500–3000 cells were seeded per well in a 96-well plate and were grown for different time points in the presence or absence of various concentrations of doxorubicin. Control cells were treated with DMSO, at the same dilution as the highest concentration of doxorubicin treatment. Luminescence was recorded with a plate reader (Promega GloMax Multi Detection System).

### Western blotting

Protein lysates were extracted using RIPA buffer (Sigma) including fresh protease and phosphatase inhibitors and standard western blotting protocol was performed as described before [62].

### Xenograft mouse model

The study consisted of 4 experimental groups each containing 10 female athymic nude mice (strain: Nude-Foxn1^nu^) after randomization. Due to the hormone dependency of the MCF7 and MCF7/LMTK3 cell lines, 17β estradiol pellets (60 day release) were implanted subcutaneously into the right flank of all participating animals 4 days prior to tumor inoculation (day −4). On day 0, 5 × 10^6^ MCF7 or GG MCF7/LMTK3 tumor cells, respectively, in 200 μl Matrigel:PBS 1:1 were implanted into the subcutaneous space of the left flank of 20 animals per cell line. Following, primary tumor sizes were measured twice weekly (Monday and Friday) by calipering. On day 10, after mean tumor volumes had reached approximately 100–200 mm^3^, animals were randomized into 2 groups per cell line, each containing 10 animals. On the same day, treatment was initiated for Groups 2 (MCF7+Dox) and 4 (MCF7/LMTK3+Dox), whereas animals of Groups 1 (MCF7) and 3 (MCF7/LMTK3) were kept untreated. Animals of Groups 2 and 4 were treated with 8 mg/kg Doxorubicin intravenously (i.v.) on days 10 and 17. Mice were sacrificed on day 28. Primary tumor tissues of all animals were photographed and collected and wet weights and volumes determined accordingly. Tumor volumes were measured every 3 or 4 days using a caliper and calculated using the formula: W^2^xL/2 (L = length and W = the perpendicular width of the tumor. Experimental protocols had been approved by the Ethics Committee for Animal Experimentation. The experimental protocol was registered by the Regierungspräsidium Freiburg (G-13/23).

### Immunofluorescence staining

Cells grown on glass coverslips were fixed in 4% paraformaldehyde for 10 min, washed in PBS and incubated with 0.3% (v/v) Triton X-100 for 10 min at RT. Following, cells were washed in PBS and blocked with 10% AB serum in PBS for 30 min. Coverslips were then incubated overnight at 4 °C with primary antibodies, diluted in the same buffer. Cells then were washed and incubated with Alexa Flour®-488 secondary anti-rabbit and Alexa Flour®-555 secondary anti-mouse antibodies (Invitrogen) at RT for 60 min. After washing, coverslips were mounted onto glass slide in Mowiol (Calbiochem) with 4′,6-diamidino-2-phenylindole (DAPI) solution [10% (w/v) Moviol, 1 µg/ml DAPI]. Coverslips were examined on a Leica TCS SP5 II STED laser scanning confocal microscope (Leica Microsystems).

### RNA extraction, library construction, and RNAseq

MCF7 cells and MCF7/LMTK3 cells were treated with 1 μM doxorubicin or DMSO for 24 h in duplicates, and total RNA was isolated using PureLinkTM RNA Mini Kit according to manufacturer’s instruction. The RNA samples were quantitated using a Qubit 2.0 (Life Technologies, Ca, USA) fluorimeter from Invitrogen with a High Sense RNA kit to obtain correct starting concentrations. Bioanalyzer was run using RNA Pico 6000 kit to determine RIN scores for the samples to assess sample quality. Each sample was diluted in order to get a final mass of 500 ng of RNA in 10 μl of RNase free water. Library prep was performed using the RNA hyper prep kit with Riboerase from KAPA Biosystems (Catalog Number KK8560) according to manufacturer’s recommendation. The library fragmentation was performed at 85 °C for 4.5 min. All Libraries underwent eight cycles of PCR with the recommended conditions. NEXTflex-96 DNA barcodes from Bio- Scientific were ligated to allow for multiplexing of samples. After library preparation, each sample was quantitated using the Qubit DNA High Sense Kit, then dilutions were performed to equalize each library to 3 ng/μl. Diluted libraries were then run on the Bioanalyzer using DNA High Sense kit to determine library molarities. Libraries were pooled which was then diluted to a final concentration of 2 nM. Pooled and diluted library was denatured and diluted for sequencing with a 1% PhiX spike-in by following the Illumina guide. Library was loaded onto the Nextseq 500 and sequencing was performed using a 75-cycle high output v2 kit.

### Comet assay

DSB repair was analysed by neutral comet assay using the Trevigen comet assay kit (4250-050-K) according to the manufacturer’s methods. 2 h after doxorubicin treatment, cells were subjected to comet analysis at the indicated time points. Cell suspensions (1 × 10^5^ cells in 50 µl PBS) were mixed with 450 µl of heated LM-Agarose at 37°C and were spread on the slides covered with agarose and allowed to solidify at 4 °C in a moist box. After a clear ring appears at edge, the slides were immersed in freshly prepared cold (4 °C) lysis solution overnight at 4 °C. The slides were removed from the lysis buffer, drained the excess buffer and transferred to an electrophoresis chamber. After equilibrated in cold 1 × neutral electrophoresis solution for 30 min, SDS-PAGE electrophoresis was conducted at 18 V for 45 min. The slides were then immersed in DNA Precipitation Solution for 30 min, followed by 70% ethanol for another 30 min at RT. The slides were drained and air-dried for more than 15 min. After staining with DAPI, comet images were captured by fluorescence microscopy. The tail moment was quantified using the ImageJ software with OpenComet plug-in [63].

### Raw data processing, alignment analysis, and identification of differentially expressed genes

High-quality clean reads were obtained after trimming the adapter sequences and removing invalid reads containing poly-N and low-quality reads from the raw data using the fastX tool kit (v 0.0.14) (http://hannonlab.cshl.edu/fastx_toolkit/license.html). The quality of reads was confirmed using the fastqc tool kit (v 0.11.5) (http://www.bioinformatics.babraham.ac.uk/projects/fastqc/). The downstream analyses were conducted using the high-quality clean reads. The clean reads were mapped to the ENSEMBL built GRCH37 using the STAR aligner (v2.5.3a) [64] with default parameters along with the ENCODE options as described in the STAR manual. We then estimated the read counts for each gene using the summarize Overlaps function provided with the R package DESeq2 [65]. Subsequently, we identified differentially expressed genes (DEGs) using DESeq2 interaction design model. The Benjamini-Hochberg corrected *P* value (*P*_adj_ ≤ 0.05) and Log2 fold change ≥ |1| were used as the threshold to screen significance of DEGs.

### Quantitative real time PCR (qRT-PCR)

Total cellular RNA was extracted from cells using the PureLink^TM^ RNA Mini Kit (Invitrogen) according to manufacturer recommendations (including DNAse treatment step). Complementary DNA synthesis reactions were performed with 2.5 ug of RNA using SuperScript III First-Strand Synthesis System (Invitrogen) according to manufacturer instruction. cDNA samples were amplified using SYBR Green PCR Master Mix on the Applied Biosystems 7500 Detection System. Each PCR reaction had three technical replicates and three biological replicates. Gene-specific forward and reverse primers are detailed in Supplementary Table 5. Furthermore, specificity and efficiency for primers were analyzed as we have previously reported [66]. The amplified transcripts were quantified using the comparative ddCT method.

### Functional annotation of DEGs

The analyses of canonical pathways, disease, and biological functions, as well as upstream regulators of DEGs was conducted using the Ingenuity Pathway (https://www.qiagenbio-informatics.com/products/ingenuity-pathway-analysis). The protein classification of the DEGs was performed using PANTHER version 11 [67]. The GO annotation analyses of gene sets were performed using www.geneontology.org [68, 69]. The protein-protein interaction analyses of gene sets were performed using the R package STRING protein-protein interaction database [70].

### Clinical specimens

Pre- and post-chemotherapy primary breast tumor pairs from 148 patients were stained for LMTK3. All patients had newly diagnosed, histologically or cytologically confirmed breast cancer and were enrolled into one of four therapeutic clinical studies that were approved by the institutional ethics committee, evaluating primary and pre-operative chemotherapy. In study 1 (*n* = 65), patients received 6 cycles of alternating sequential doxorubicin (A) and docetaxel (T) 3 weekly and were randomized to start either with doxorubicin or docetaxel (A →T→A→T→A→T or T→A→T→A→T→A). In study 2 (*n* = 31), patients were treated with 4 cycles of docetaxel modulated with oral ketoconazole, 3 weekly. In study 3 (*n* = 25), patients were randomized to 4 cycles of doxorubicin or 4 cycles of docetaxel, 3 weekly. In study 4 (*n* = 27), patients were treated with 4 cycles of doxorubicin and cyclophosphamide, 3 weekly, with or without low-dose sunitinib. Tumor biopsies were taken at baseline and after cycle 1 chemotherapy in all four studies; one additional tumor biopsy after 4 cycles of chemotherapy was obtained in studies 1 and 4. Written informed consent was obtained from all subjects enrolled into these studies 1–4. The ethics committee that approved the study conduct was the National Healthcare Group Domain Specific Review board. Clinical trial registration numbers: study 1 (NCT00212082), study 2 (NCT00212095), study 3 (NCT00669773), study 4 (NCT02790580).

### Immunohistochemistry

Immunohistochemistry staining for LMTK3 was performed and nuclear staining was scored as previously described [1, 5]. Paired t test was applied to determine the difference in LMTK3 expression between matched pre and post-treatment tumors. All statistical analyses were performed using the IBM SPSS package (version 19.0 for Windows, IBM SPSS Inc., USA) with significance set at the 5% level.

### Statistics

All data are expressed as means ± standard error of the mean (SEM). Data were analysed by paired Student’s *t*-test and/or two-way ANOVA test, using GraphPad Prism 7.00 (GraphPad Software, Inc., San Diego, USA).

## Electronic supplementary material

Supplementary Figures(PDF 934 kb)

Supplementary Tables(PDF 330 kb)

Supplementary Excel file Legends(PDF 290 kb)

Supplementary Excel Files (1-4)(XLSX 12686 kb)

Supplementary Excel Files (5-13)(XLSX 409 kb)
