# Supplementary material for: LMTK3 confers chemo-resistance in breast cancer
Source: Oncogene. 2018 Mar 15;37(23):3113–30. doi: 10.1038/s41388-018-0197-0 (PMC5992129; doi:10.1038/s41388-018-0197-0)
Supplement: Supplementary file 1 — Supplementary Figures(PDF 934 kb) [file 41388_2018_197_MOESM1_ESM.pdf]

Supplementary Figure 1

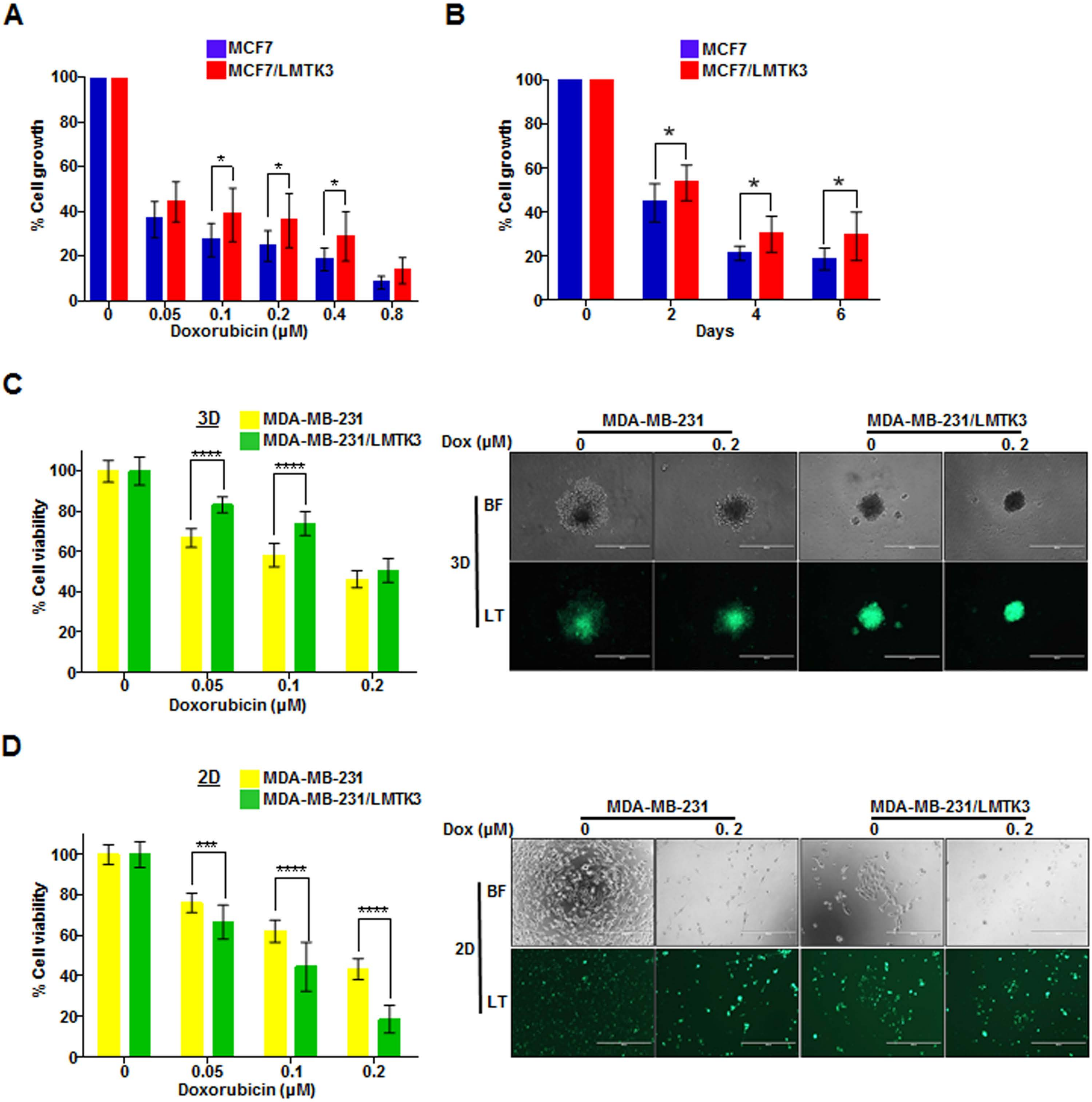

Supplementary Figure 2

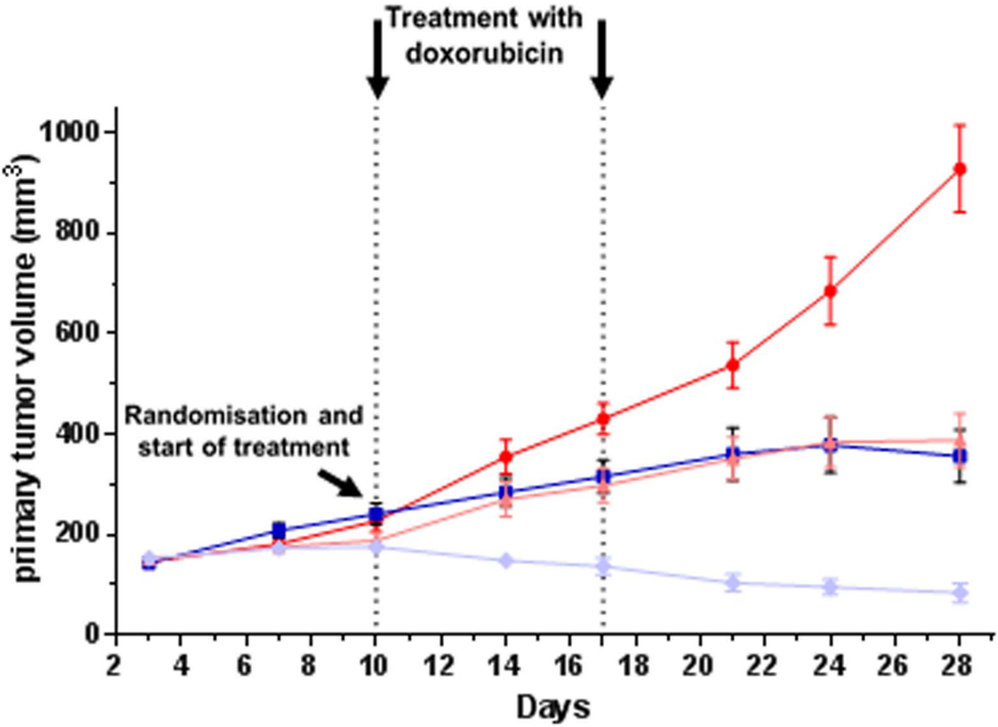

Group 1: 5x10^6 MCF7 cells implanted sc. on day 0 (n=10)

Group 2: 5x10^6 MCF7 cells cells implanted sc. on day 0;
8mg/kg Doxorubicin i.v. on days 10 and 17 (n=10/7)

Group 3: 5x10^6 MCF7/LMTK3 cells implanted sc. on day 0 (n=10)

Group 4: 5x10^6 MCF7/LMTK3 cells implanted sc. on day 0;
8mg/kg Doxorubicin i.v. on days 10 and 17 (n=10/9)

Supplementary Figure 3

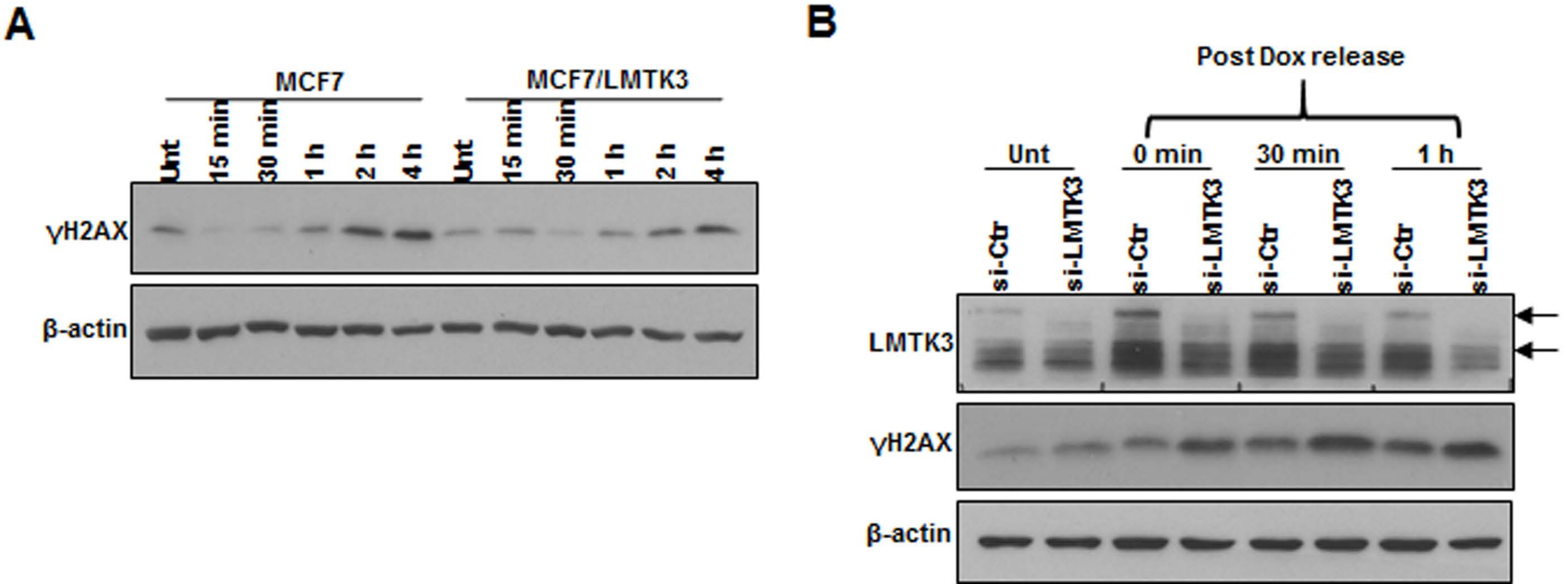

Supplementary Figure 4

Principal Component Analysis & Sample Clustering Plot Assessing Overall Similarity Between Samples

A

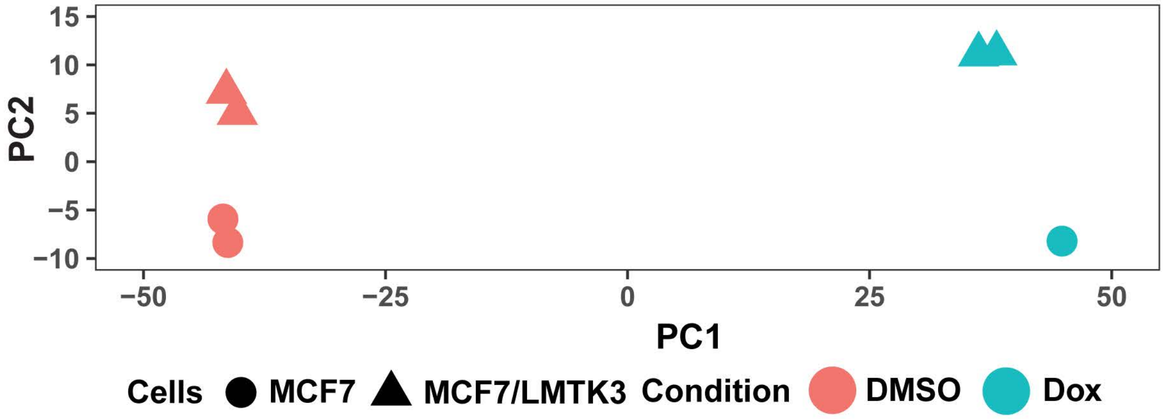

B

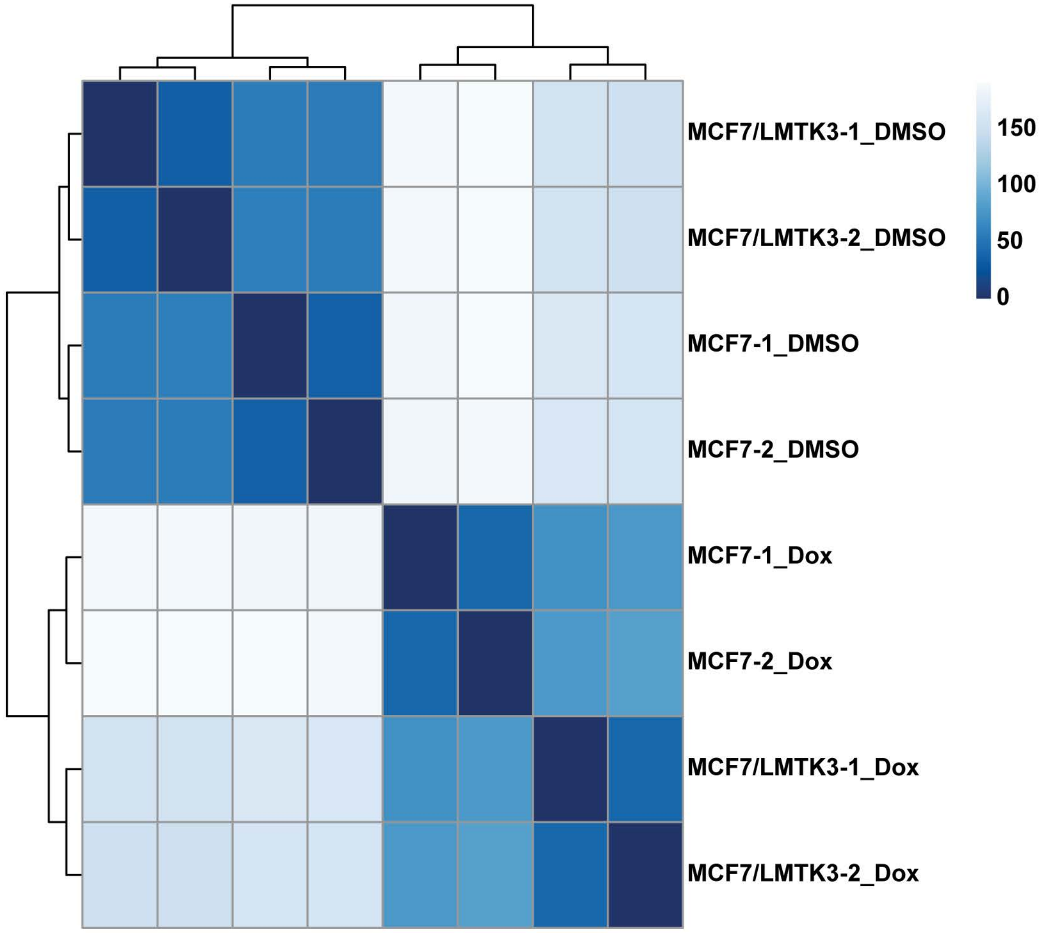

Supplementary Figure 5

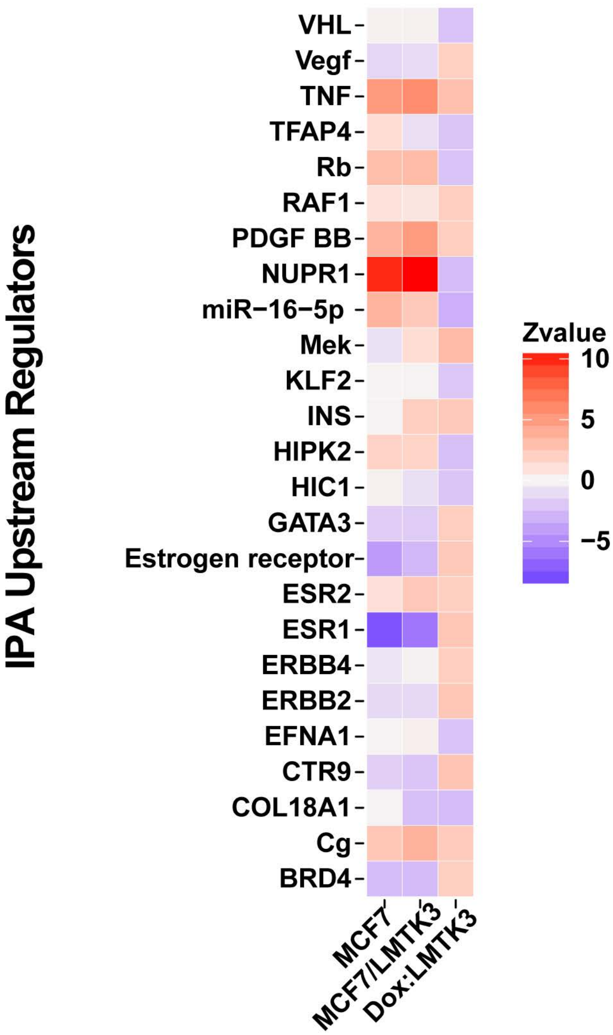

Supplementary Figure Legends

Supplementary Figure 1. LMTK3 over-expression decreases doxorubicin-induced cytotoxicity.

The percentage (%) of growth was evaluated by SRB assay in MCF7 and MCF7/LMTK3 cells, following treatment with: **(A)** different concentrations of doxorubicin (0.05, 0.1, 0.2, 0.4 and 0.8 μM) for 6 days; **(B)** 0.8 μM of doxorubicin for different time points (2, 4, 6 days).
Following treatment with different concentrations of doxorubicin (0.05, 0.1 and 0.2 μM) for 72 h, the percentage (%) of cell viability, was assessed by CellTiter-Glo assay in MDA-MB-231 and MDA-MB-231/LMTK3 cells cultured in either **(C)** 3D (spheroids) or **(D)** 2D (monolayers). All error bars represent the mean ± the SD from 3 independent experiments (* P ≤ 0.05, *** P ≤ 0.001, **** P ≤ 0.0001).

Supplementary Figure 2. *In vivo* monitoring of xenograft tumor growth.

MCF7 (Groups 1-2) and MCF7/LMTKTK3 (Groups 3-4) tumor cells, respectively, were implanted s.c. on day 0. After randomization on day 10, treatment with Doxorubicin was initiated in case of Groups 2 and 4, whereas Groups 1 and 3 were kept untreated. During the course of the study, the growth of the subcutaneously implanted primary tumors was determined twice weekly by caliper measurement. Data are displayed as means ± SEM.

Supplementary Figure 3. LMTK3 levels affect γH2AX phosphorylation following doxorubicin treatment.

(A) MCF7 and MCF7/LMTK3 cells were treated with 1 μM of doxorubicin for different time points and Western blot analysis for endogenous γH2AX levels was performed. **(B)** MCF7 cells were transfected with either LMTK3 small interfering RNA (siRNA) (siLMTK3) or with a non-targeting control siRNA (siControl) for 72 h. Cells were then treated with 1 μM of doxorubicin. After 2 h of treatment, cells were washed and cultured in complete media. Cells were harvested at the indicated times and analysed by Western blot with the respective antibodies. β-actin was used as loading control.

Supplementary Figure 4. (A) Principal Component Analysis (PCA) performed using DESeq2 rLog-normalized RNAseq data. Loadings for principal components 1 (PC1) and PC2 are reported in the graph. **(B)** Hierarchical clustering analyses performed using DESeq2 rLog-normalized RNAseq data. Color code from white to dark blue refers to the distance metric used for clustering, with dark blue referring to maximum of correlation values.

Supplementary Figure 5. Heatmaps comparing Z scores of upstream regulators whose downstream target genes were differentially regulated (Z score ≥ |2.0| & P≤ 0.05) by doxorubicin in MCF7 and MCF7/LMTK3 cells. The significance P values were calculated by Fisher's exact test. The activation or inhibition of the canonical pathways and disease bio functions is defined by Z score. A Z score of ≥2 is considered as significant activation and a Z score of ≤-2 is considered as significant inhibition. The Z score between (0,2) or (-2,0) represents trend towards activation or inhibition respectively.
