# Supplementary material for: LMTK3 confers chemo-resistance in breast cancer
Source: Oncogene. 2018 Mar 15;37(23):3113–30. doi: 10.1038/s41388-018-0197-0 (PMC5992129; doi:10.1038/s41388-018-0197-0)
Supplement: Supplementary file 2 — Supplementary Tables(PDF 330 kb) [file 41388_2018_197_MOESM2_ESM.pdf]

Supplementary Tables

Supplementary Table 1

		γH2AX foci count	
Dox treatment	Time	**MCF7**	**MCF7/LMTK3**
	30min	14.66 ± 1.25	14.44 ± 1.02
	60min	22.90 ± 2.09	20.30 ± 1.67
	90min	36.98 ± 1.58	30.24 ± 1.53
	120min	51.42 ± 1.64	35.98 ± 1.57

		γH2AX foci count	
Post Dox treatment	Time	**MCF7**	**MCF7/LMTK3**
	30min	45.77 ± 2.01	41.84 ± 1.79
	60min	40.88 ± 1.81	48.58 ± 1.48
	90min	41.20 ± 1.25	41.10 ± 2.26
	120min	42.00 ± 1.60	36.34 ± 1.45
	240 min	40.60 ± 1.44	34.46 ± 1.50
	600 min	24.44 ± 1.35	18.32 ± 1.15

Supplementary Table 1. LMTK3 over-expression impedes doxorubicin-induced γH2AX phosphorylation.

MCF7 and MCF7/LMTK3 cells were treated with 1 µM of doxorubicin for 2 h and incubated for another 10 h in fresh complete media. Quantitative analysis of γH2AX foci for different time points was performed.

Supplementary Table 2

Time	% Tail DNA	
	**MCF7**	**MCF7/LMTK3**
Untreated	1.67 ± 0.26	1.85 ± 0.28
6 h (post treatment)	21.71 ± 1.35	12.89 ± 1.10
24 h (post treatment)	12.10 ± 1.00	8.49 ± 0.80

Supplementary Table 2. LMTK3 over-expression results in fewer DNA tail moments.

Cells were treated with 1 µM of doxorubicin for 2 h to induce DNA breaks. Following drug treatment, the media was removed and cells were incubated with fresh complete media for another 6 h and 24 h. Quantitative analysis of % tail DNA for different time points was performed.

Supplementary Table 3.

Targets	*RNAseq*		*RT-PCR validation*		
	MCF7 (Doxo 0.5ug/ml)[1]	*p-adj*[2]	*MCF7 (Doxo 0.5ug/ml)*[3]	*Std. Error*[4]	*p-value*[5]
LMTK3	1.12	8.17E-01	2.54	0.34	1.05E-02
POLG2	5.21	1.21E-123	2.28	0.24	6.13E-03
XIST	-1.63	3.25E-13	-1.02	0.45	1.10E-02
RPS19	2.41	3.04E-112	2.70	0.19	7.81E-04
CNR1	192.67	2.68E-28	53.84	7.15	1.79E-03
SOX6	-5.66	1.12E-07	-6.80	1.57	7.58E-03
HEY1	3.16	3.66E-04	3.69	1.09	6.95E-02
STMN1	-1.88	3.31E-37	-16.30	5.49	3.44E-02
NET1	-5.24	1.04E-231	-6.56	0.90	1.08E-03
HMGB2	-1.92	1.65E-34	-0.44	0.07	3.64E-05
KCNJ8	-504.95	3.48E-09	-47.20	14.10	2.68E-02
CYP1A1	44.32	6.24E-18	5.21	0.58	1.96E-03
H1F0	-5.31	5.19E-241	-2.49	0.24	1.25E-04
TFAP2C	1.99	1.44E-61	3.16	0.16	1.70E-04

Supplementary Table 3. qRT-PCR validation of RNAseq data in MCF7 cells. RNAseq: [1]Average fold expression change (n=2) between MCF7 (Doxo 0.5ug/ml) and MCF7 (DMSO); [2]*P*-value corrected adjusted using *Benjamini-Hochberg procedure.* RT-PCR validation:[3]Average fold expression change (technical replicates=3; biological replicates= 3) between MCF7 (Doxo 0.5ug/ml) and MCF7 (DMSO); [4]Standard Error of mean calculated for n = 3; [5]*P*-value showing significance of fold expression changes calculated by RT-PCR.

Supplementary Table 4.

Targets	*RNAseq*		*RT-PCR validation*		
	MCF7/LMTK3 (Doxo 0.5ug/ml)[1]	*p-adj*[2]	*MCF7/LMTK3 (Doxo 0.5ug/ml)*[3]	*Std. Error*[4]	*p-value*[5]
LMTK3	1.42	0.000415112	0.10	0.10	8.99E-04
POLG2	1.36	4.69E-05	1.79	0.81	3.81E-01
XIST	3.27	0.434538878	3.80	3.18	5.47E-01
RPS19	1.95	4.43E-66	2.00	0.19	5.83E-03
CNR1	190.02	2.48E-12	34.07	8.16	1.54E-02
SOX6	4.06	1.40E-42	4.84	0.25	9.79E-05
HEY1	-2.16	0.022866463	1.68	0.29	7.77E-02
STMN1	-2.69	4.21E-86	-6.87	1.31	3.89E-03
NET1	-1.77	1.18E-32	-5.00	0.83	1.98E-03
HMGB2	-2.93	9.02E-88	0.75	2.08	9.11E-01
KCNJ8	-13.09	6.25E-31	-308.24	210.55	2.16E-01
CYP1A1	3.56	2.88E-21	8.39	4.92	2.07E-01
H1F0	-1.93	6.18E-43	-5.79	1.88	2.24E-02
TFAP2C	2.55	2.25E-113	1.73	0.24	4.07E-02

Supplementary Table 4. qRT-PCR validation of RNAseq data in MCF7/LMTK3 cells. RNAseq: [1]Average fold expression change (n=2) between MCF7/LMTK3 (Doxo 0.5ug/ml) and MCF7/LMTK3 (DMSO); [2]*P*-value corrected adjusted using *Benjamini-Hochberg procedure.* RT-PCR validation:[3]Average fold expression change (technical replicates=3; biological replicates= 3) between MCF7/LMTK3 (Doxo 0.5ug/ml) and MCF7/LMTK3 (DMSO); [4]Standard Error of mean calculated for n = 3; [5]*P*-value showing significance of fold expression changes calculated by RT-PCR.

Supplementary Table 5.

Gene	Sequence
SOX6-FP	GTCACTTTCATGAATTCAAAGGCA
SOX6-RP	GCTTGCTTGGAAGACATTCTTAAAT
HEY1-FP	TAATTGAGAAGCGCCGACGA
HEY1-RP	GCTTAGCAGATCCTTGCTCCA
KCJN8-FP	GGAGTCCACTGTGTGTGTGA
KCJN8-RP	AAAACCGTGATGGCCAAAGG
CYP1A1-FP	CCCCACAGCACAACAAGAGA
CYP1A1-RP	CAGGGGTGAGAAACCGTTCA
H1F0-FP	TCAAGCAGACCAAAGGGGTG
H1F0-RP	TTTGGCGTGGCTACCTTCTT
NET1-FP	GAGCTGGACGGGAGATGTTC
NET1-RP	GGCTCCTTCAGATCAAGGCT
HMGB2-FP	CTCCAATACCCTCGGGTGGA
HMGB2-RP	GCGTACGAGGACATTTTGCC
STMN1-FP	CCCACGAGGGTTCTTCTGTC
STMN1-RP	ACTGGGATAAGGAAAGTCCTGC
POLG2-FP	CTTCACCCTTGTTTAGCCCCT
POLG2-RP	AGCCCTTGACAAACCTGTCT
RPS19-FP	AGTCCCCGAATGGGTGGATA
RPS19-RP	CGCTGTGGAAGCAGCTCG
TFAP2C-FP	GAAGAGGACTGCGAGGATCG
TFAP2C-RP	GCTGATATTCGGCGACTCCA
NR4A2-FP	CTCCCAGAGGGAACTGCAC
NR4A2-RP	GCCTGTCCAATCTCCTCCTTAG
LMTK3-FP	AATGTCTGCGTAACCGCACG
LMTK3-RP	GGCGAATCCATCGGGGTG
XIST-FP	GACACAAGGCCAACGACCTA
XIST-RP	TCGCTTGGGTCCTCTATCCA
CNR1-FP	TCGTTCTAGCGGACAACCAG
CNR1-RP	AATCTCTTTGCCCCTTCGCA

Supplementary Table 5. Sequences of primers used for the qRT-PCR validation of RNA-Seq data. FP: Forward primer; RP: Reverse primer.
