# Supplementary material for: LMTK3 confers chemo-resistance in breast cancer
Source: Oncogene. 2018 Mar 15;37(23):3113–30. doi: 10.1038/s41388-018-0197-0 (PMC5992129; doi:10.1038/s41388-018-0197-0)
Supplement: Supplementary file 3 — Supplementary Excel file Legends(PDF 290 kb) [file 41388_2018_197_MOESM3_ESM.pdf]

Supplementary Excel file Legends

Supplementary Excel File 1: DESeq2 report showing differentially expressed genes for comparison model: Doxorubicin treated vs DMSO treated MCF7 cells. [1]Ensembl ID; [2]baseMean: Mean of the normalized read counts across all the samples; [3]Log2 fold change: Log2 fold change is the effect size estimate that measures how much gene's expression have changed due treatment with doxorubicin treatment in comparison to treatment with DMSO; [4]lfcSE: Standard error estimate for the Log2 fold change estimate; [5]stat: Wald-statistics to test whether the data provides sufficient evidence to conclude if the particular effect size estimate value is really different from zero; [6]P value: indicates the probability that a fold change as strong as the observed one, or even stronger, would be seen under DMSO treatment; [7]padj: Benjamini-Hochberg (BH) corrected P values. BH method calculates an adjusted P value for each gene which answers the following question: if we called significant all genes with a P value less than or equal to this gene's P value threshold, what would be the fraction of false positives among them; [8]symbol: Gene symbol; [9]entrez: entrez id of each gene.

Supplementary Excel File 2: DESeq2 report showing differentially expressed genes for comparison model: Doxorubicin treated vs DMSO treated MCF7/LMTK3 cells.

Supplementary Excel File 3: DESeq2 report showing the variation in the normalized read counts of each gene across all the samples. The deviation from mean variation for each gene was plotted in the heatmap (Figure 1D).

Supplementary Excel File 4: DESeq2 report showing differentially expressed genes from the interaction model. These genes respond differentially to doxorubicin treatment across MCF7 and MCF7/LMTK3 cells.

Supplementary Excel Files 5–7: Files showing the Ingenuity Pathway Analysis (IPA) tool identified upstream regulators that can explain the observed gene expression changes in the doxorubicin treated MCF7 (File 5), MCF7/LMTK3 (File 6), and Dox:LMTK3 genes (File 7). [1]Upstream Regulators: Upstream molecules identified by ingenuity pathway analysis; [2]Expr Log ratio: Log2 fold change as

identified by DESeq2 analysis that measures how much gene's expression have changed due treatment with doxorubicin treatment in comparison to treatment with DMSO; [3]Molecule type: Type of upstream molecule; [4] Predicted Activation State: The ingenuity upstream regulator analysis is based on knowledge from the literature of expected effects between transcriptional regulators and their target genes stored in the Ingenuity® Knowledge Base. The analysis examines how many known targets of each transcription regulator are present in the doxorubicin treated MCF7 (File 5), MCF7/LMTK3 (File 6), and Dox:LMTK3 genes (File 7), and also compares their direction of change (i.e. expression in the doxorubicin treated relative to DMSO) to what is expected from the literature in order to predict likely relevant transcriptional regulators. If the observed direction of change is mostly consistent with a particular activation state of the transcriptional regulator ("activated" or "inhibited"), then a prediction is made about that activation state; [5]Activation Z score: The sign of the calculated Z score reflects the overall predicted activation state of the regulator (<0: inhibited, >0: activated). In practice, the Z score greater than 2 or smaller than -2 can be considered significant; [6]P value of overlap: Fishers-Exact Test is used to calculate the P value significance that signifies whether there is a statistically significant overlap between the dataset genes and the genes that are regulated by the upstream regulator; [7]Target Molecule in Dataset: Molecules identified to be regulated by the upstream regulator and are enriched in the dataset.

Supplementary Excel File 8: PANTHER protein class analysis on Dox:LMTK3 genes. [1]Protein Class: Category of proteins and their accession numbers; [2]No. of genes: number of proteins identified from the list of Dox:LMTK3 genes to be of a particular category of protein; [3]hit against total: Percentage of gene hits against the total.

Supplementary Excel File 9: File comparing the Z score (activation/inhibition) of the canonical pathways identified by the IPA between doxorubicin treated MCF7 and MCF7/LMTK3.

Supplementary Excel File 10: File comparing the Z score (activation/inhibition) of disease and bio functions identified by the IPA between doxorubicin treated MCF7 and MCF7/LMTK3.

Supplementary Excel Files 11–13: Files 11, 12, and 13 show the GO ontology identified biological processes enriched for genes from clusters 1–3 respectively. The clusters were identified using the protein-protein interaction clustering analysis on Dox:LMTK3 genes. [1]GO biological process complete: Identified bioprocesses; [2]No. of Homo Sapiens (REF): Total number of genes in the reference (homo sapiens genome) annotated to a particular GO biological process; [3]No. of enriched from upload: No. of genes from the uploaded dataset (listed beside the table) found to be enriched in the GO biological process; [4]Expected: Number of genes expected based on size of gene list and number of genes in genome with GO term; [5]Fold enrichment: Number of genes found in the gene list with a certain GO term divided by value in Expected column; [6]+/-: The symbols indicate overrepresentation or underrepresentation respectively; [7]P value: This value is obtained using the Fisher exact test and gives the probability or chance of seeing at least x number of genes out of the total n genes in the list annotated to a particular GO biological process, given the proportion of genes in the whole genome that are annotated to that GO biological process.
